# Gastrectomy promoted diabetes remission involves the molecular clock and epigenetic mechanisms in a rat model of lean type 2 diabetes

**DOI:** 10.1038/s41598-025-29273-y

**Published:** 2025-12-08

**Authors:** Aurélie Le Lay, François Brial, Claude Rouch, Xiaojian Shao, Mathieu Bourgey, Kazuhiro Sonomura, Huiting Ou, Sara Ghezzal, Mylène Vincent, Marylène Rugard, Karine Audouze, Jiannis Ragoussis, Fumihiko Matsuda, Guillaume Bourque, Elin Grundberg, Mark Lathrop, Christophe Magnan, Dominique Gauguier

**Affiliations:** 1 Functional and Adaptive Biology Unit, CNRS UMR 8251, University Paris Cité, 4 Rue Marie Andrée Lagroua Weill-Halle, 75013 Paris, France; 2 INSERM U1132 Biologie de L’os Et du Cartilage (BIOSCAR), University Paris Cité, 75010 Paris, France; 3 Victor Phillip Dahdaleh Institute of Genomic Medicine, McGill University, 740 Doctor Penfield Avenue, Montreal, QC H3A 0G1 Canada; 4 Digital Technologies Research Centre, National Research Council Canada, 1200 Montreal Road, Ottawa, ON K1A 0R6 Canada; 5 Life Science Research Center, Technology Research Laboratory, Shimadzu Corporation, Kyoto, 604-8511 Japan; 6 Center for Genomic Medicine, Graduate School of Medicine, Kyoto University, Kyoto, 606-8507 Japan; 7 University Paris Cité, INSERM UMR 1124, 45 Rue Des Saint-Pères, 75006 Paris, France; 8 Department of Pediatrics, University of Missouri-Kansas City School of Medicine, 2411 Holmes Street, Kansas City, MO 64108 USA; 9 Genomic Medicine Center, Children’s Mercy Kansas City, 2401 Gillham Road, Kansas City, MO 64108 USA

**Keywords:** Epigenome, Goto-Kakizaki rat, Bariatric surgery, Circadian clock, Lipidome, Metabolome, Transcriptome, Physiology, Diseases

## Abstract

Bariatric surgery results in type 2 diabetes (T2D) improvement. To identify mechanisms associated with gastrectomy-promoted T2D remission in lean individuals, we performed pathophysiological, behavioural and molecular (liver transcriptome, metabolome and lipidome) investigations in the Goto-Kakizaki (GK) model of spontaneously-occurring non-obese T2D following vertical sleeve gastrectomy (VSG) or sham operation. VSG resulted in sustained reduction in hyperglycemia and changes in nycthemeral feeding patterns and activity. Liver transcriptome and lipidome profiling pointed to changes in the expression of genes involved in inflammation, PPAR signalling and fatty acid metabolism, and in the regulation of phosphatidylcholine and lysophosphatidylethanolamine classes. Deeper analysis revealed altered expression of genes involved in histone methylation and co-ordinately differential transcription of key regulators of the molecular clock (*Clock*, *Arntl/Bmal1*, *Per1*, *Per2*, *Per3*). In addition to previously reported changes in bile acid metabolism and gut microbiome in this model of VSG, our findings underline the multiple biological mechanisms associated with diabetes remission following VSG and suggest a contribution of chronobiology and epigenetic processes in the long-term therapeutic consequences of VSG in the context of polygenic non-obese T2D.

**Supplementary Information:**

The online version contains supplementary material available at 10.1038/s41598-025-29273-y.

## Introduction

Type 2 diabetes (T2D) and obesity are characterised by an etiology combining both environmental influences and genetic risk factors^1^. Bariatric surgery, which is routinely used in obese patients, results in sustained weight loss^2,3^ but also in improved glucose homeostasis, through mechanisms that are at least in part independent of weight loss and reduced caloric intake^4^. Roux-en-Y gastric bypass (RYGB), vertical sleeve gastrectomy (VSG) and stomach sparing protocols (e.g. duodenal-jejunal and jejuno-ileal bypass, jejunectomy) have consistently demonstrated their beneficial role in T2D patients^5^, but the biological processes involved remain poorly understood. Changes in the microbiome architecture and bile acids, improved insulin sensitivity and restored β-cell mass and function mediated by incretins, including the glucagon like peptide (GLP1), collectively account for instances of T2D remission after bariatric surgery^6^. However, the combination of obesity and T2D in patients undergoing bariatric surgery often prevents the separation of biological mechanisms that directly lead to restored glycemic control to those that are secondary to weight loss.

Preclinical models maintained in controlled conditions (diet, light/dark cycle) have been used to investigate biological consequences of bariatric surgery^7^. The Goto-Kakizaki (GK) rat strain has several advantages over many preclinical models used in gastrectomy experiments, which often combine diabetes and obesity. It is highly inbred and spontaneously develops the main features of T2D (β-cell mass depletion, impaired insulin secretion, insulin resistance) in the absence of obesity, following genomic enrichment in naturally-occurring genetic polymorphisms over many generations of successive breeding of glucose intolerant outbred rats^8^. Even though diabetes in the GK strain is caused by many genes that have been localised in the rat genome by linkage mapping and functional genomics (Reviewed in^9^)^10^, and directly or indirectly leading to multiple pathological features, bariatric surgery is able to improve glycemic control in this strain^11–13^. We showed that reduction of hyperglycemia by gastrectomy in GK rats involves alterations in gut microbiota architecture and bile acid metabolism^14^, and that surgical disruption of neuronal connections between the central nervous system and pancreatic islets and the gastrointestinal tract has little impact on regional brain transcriptional regulation^15^. These findings, alongwith reports of improved hepatic insulin sensitivity and suppression of hepatic gluconeogenesis following bariatric surgery in GK and fat fed rats^16,17^, suggest that changes in liver function play a central role in gastrectomy-promoted improvement of glucose homeostasis.

To characterise further the physiological and hepatic molecular mechanisms contributing to VSG-promoted T2D remission in an experimental model of T2D devoid of obesity, we carried out detailed analyses of nycthemeral feeding patterns and activity in gastrectomized GK rats and sham operated GK controls, followed by profiling of liver transcriptome, metabolome and lipidome. Our findings suggest that hepatic molecular mechanisms relevant to chronobiology and epigenetic processes are involved in VSG-promoted long term improvement in glucose homeostasis in this preclinical model of T2D in the absence of confounding effects of weight loss. They provide advanced knowledge for the development of non-surgical strategies for the treatment of T2D and obesity.

## Results

### VSG in lean diabetic GK rats results in sustained improvement in glycemic regulation and reduced food intake

**Fig. 1:**
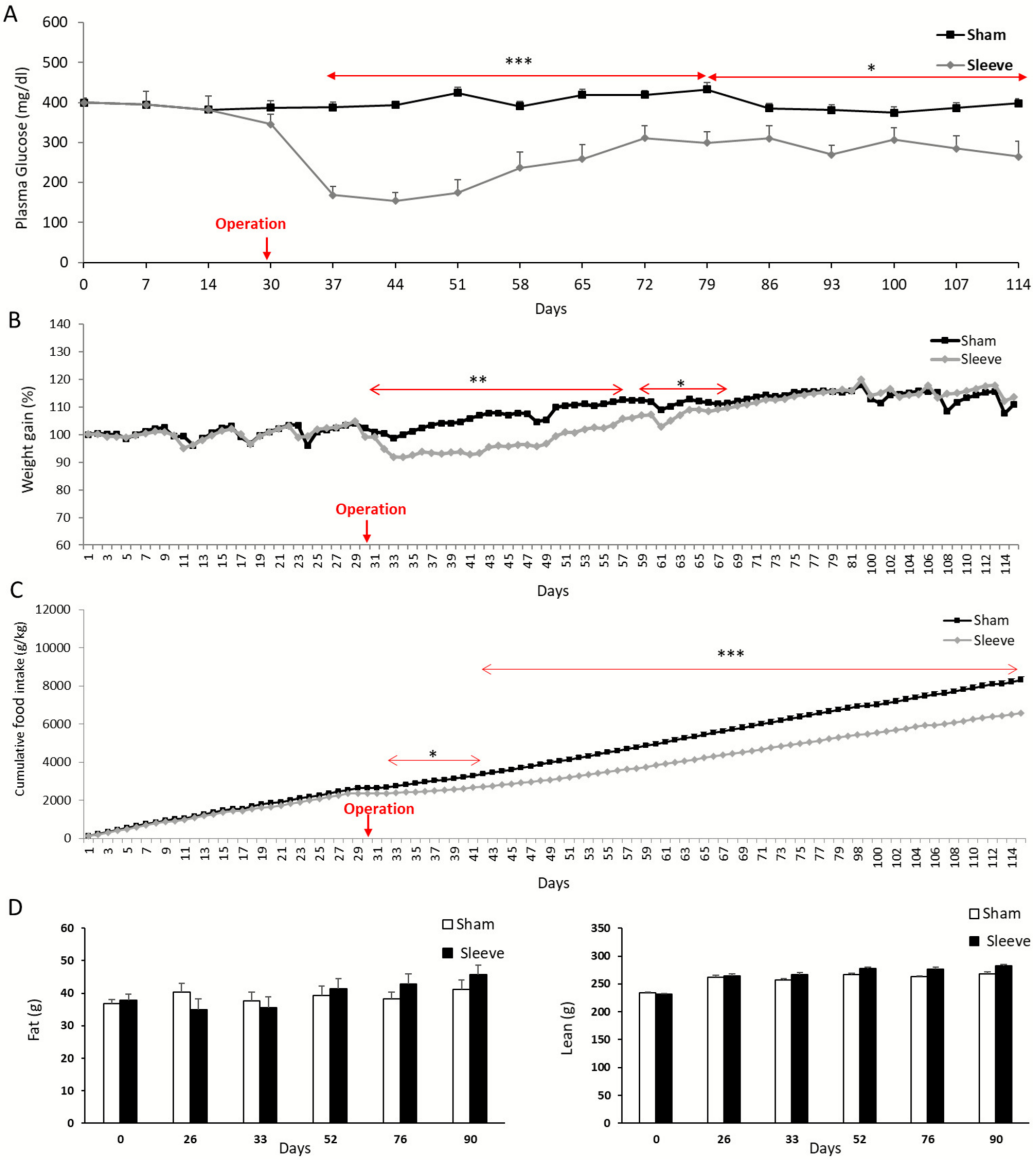
Follow up analysis of glycemia, body weight and food intake in gastrectomized and sham operated GK rats. Blood glucose was monitored weekly in free fed rats prior to VSG or sham operation at day 30, and over a period of 84 days afterwards (**A**). Body weight (**B**) and food intake (**C**) were measured daily over the same period. Fat mass and lean mass (**D**) were determined on the day of surgery (D0) and 26, 33, 52, 76 and 90 days afterwards. Results are means ± SEM. ***p < 0.005, **p < 0.01, *p < 0.05 significantly different between VSG (n = 6) and sham operated (n = 6) animals.

To verify our previous report of VSG-promoted improvement of glucose homeostasis in our GK colony^14^, a new batch of GK rats underwent VSG or sham operation. Blood glucose dropped within a week after VSG and remained significantly lower than in sham operated GK rat over a period of 80 days until the end of the experiment (Fig. 1A). Body weight and food intake also rapidly dropped after VSG but gastrectomized GK rats recovered the normal body weight of lean sham operated GK rats 38 days after surgery (Fig. 1B,C). In contrast, cumulative food intake remained significantly lower in VSG rats than in sham-operated rats until the end of the experiment (Fig. 1C). This effect is primarily driven by the pronounced early postoperative hypophagia commonly observed after VSG in many rodent studies, and progressively attenuates over subsequent weeks^18^. Fat mass and lean mass were similar in gastrectomized and sham-operated GK rats on the day of operation and over the 90 following days (Fig. 1D).

### VSG results in changes in nycthemeral feeding patterns and activity in GK rats

**Fig. 2:**
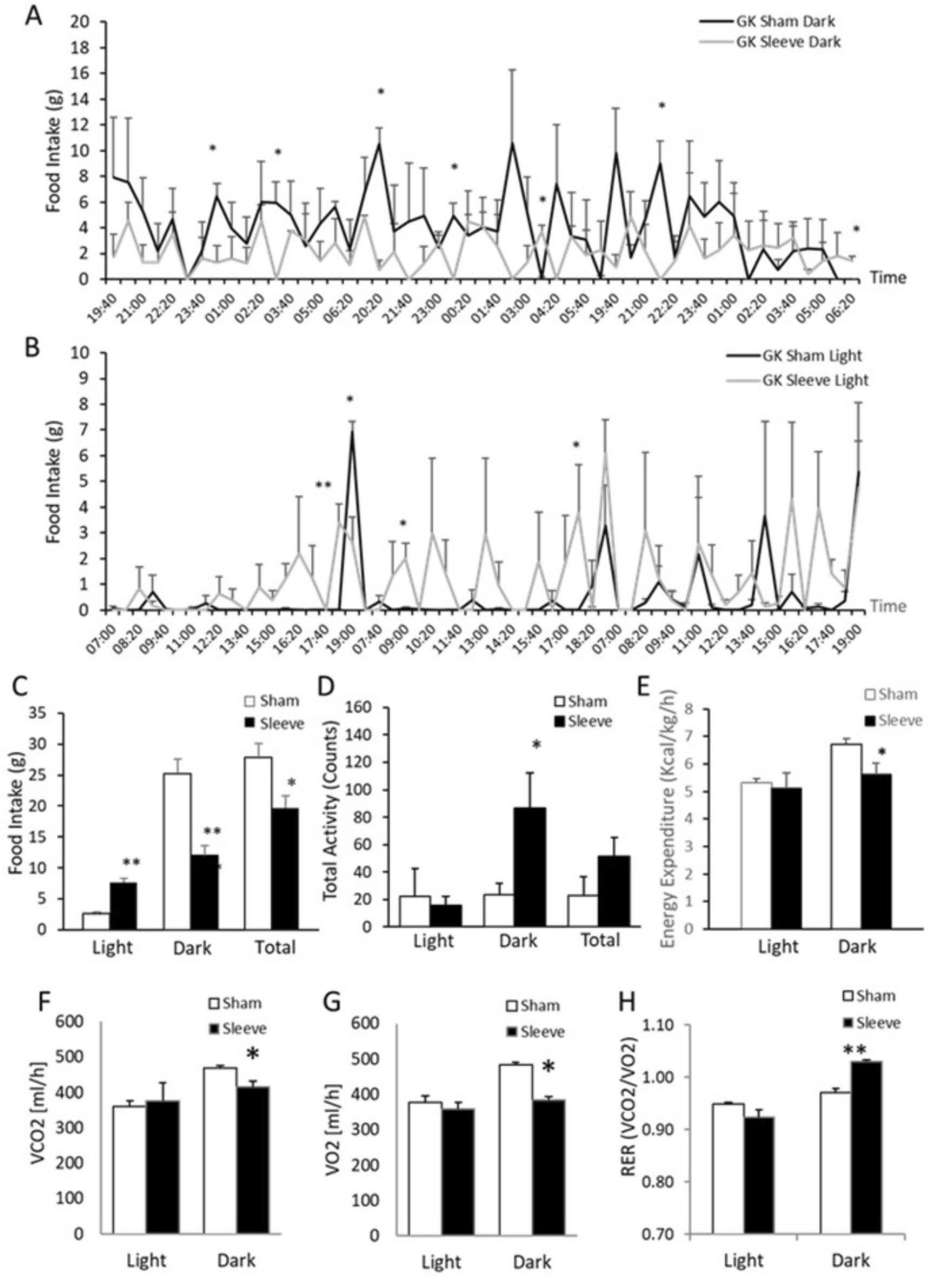
Nycthemeral feeding patterns and activity, and energy expenditure following vertical sleeve gastrectomy (VSG) or sham operation in male GK rats. Three months after bariatric surgery, spontaneous nycthemeral feeding was recorded every 80 min over a period of three days (**A**, **B**, **C**) and diurnal and nocturnal activities were recorded (**D**) (n = 6 per group). Food intake (**C**), total activity (**D**), energy expenditure (**E**), VCO_2_ (F), VO_2_ (**G**) and respiratory exchange ratio (RER) (**H**) were determined in VSG and sham rats (n = 6 per group). Results are means ± SEM. ***p < 0.005, **p < 0.01, *p < 0.05 significantly different between VSG and sham operated rats.

Growing evidence supports a role of chrono-nutrition and temporal eating patterns in metabolic health^19^. To test the hypothesis of an involvement of chronobiology in VSG-promoted improvement of glucose homeostasis, nycthemeral measures of food intake recorded every 80 min over a period of three days, activity and respiratory exchange ratio (RER) (VCO_2_/VO_2_) were determined in gastrectomized and sham operated GK rats. As expected, sham-operated GK rats exhibited predominant nocturnal pattern of food consumption over the three days of recording (Fig. 2A) and no food consumption during the light phase, with the exception of the light–dark transition period (Fig. 2B). In contrast, during the same period of recording, gastrectomized GK rats showed a similar pattern of feeding during the dark phase (Fig. 2A) and the light phase (Fig. 2B). Overall, over the three days of recording of food intake, the total consumption of food in VSG GK rats was increased during the light phase and decreased during the dark phase when compared to sham operated rats (Fig. 2C). VSG GK rats showed an almost even consumption of food during the light and dark phases (Fig. 2C). Nevertheless, total activity during the light phase was similar in VSG GK rats and in sham operated controls (Fig. 2D). In contrast, the significant changes in nycthemeral feeding patterns in GK rats following VSG were associated with increased nocturnal activity when compared to sham operated controls (Fig. 2D). Energy expenditure was similar in the two groups during the light phase, but significantly decreased during the dark phase in VSG GK rats when compared to sham operated GK rats (Fig. 2E). Both VCO_2_ (Fig. 2F) and VO_2_ (Fig. 2G) were significantly decreased in VSG GK rats during the dark phase leading to a significant increase in RER in VSG GK when compared to sham rats (Fig. 2H).

These results support the involvement of mechanisms relevant to chronobiology in response to gastrectomy and improved glucose homeostasis.

### Liver transcriptome is deeply altered following VSG-promoted reduction of glucose intolerance in GK rats

Owing to the known impact of bariatric surgery on hepatic insulin sensitivity and bile acid metabolism^6^, which we have previously documented in gastrectomized GK rats^14^, and the role of the liver in the regulation of the peripheral circadian clock^20^, we hypothesized that altered hepatic gene expression may explain nycthemeral behavioral changes in feeding patterns and improved glucose homeostasis and liver histopathology following VSG in GK rats. Liver mRNA sequencing in gastrectomized and sham-operated GK rats identified a total of 3102 genes differentially expressed between the two rat groups, including 242 sequences corresponding to gene models, predicted genes and non-coding RNA (Supplementary Table S1). There were equivalent numbers of down-regulated and up-regulated genes in response to VSG.

### Liver transcriptome profiling underlines the profound impact of VSG in GK rats on multiple biological mechanisms

**Fig. 3:**
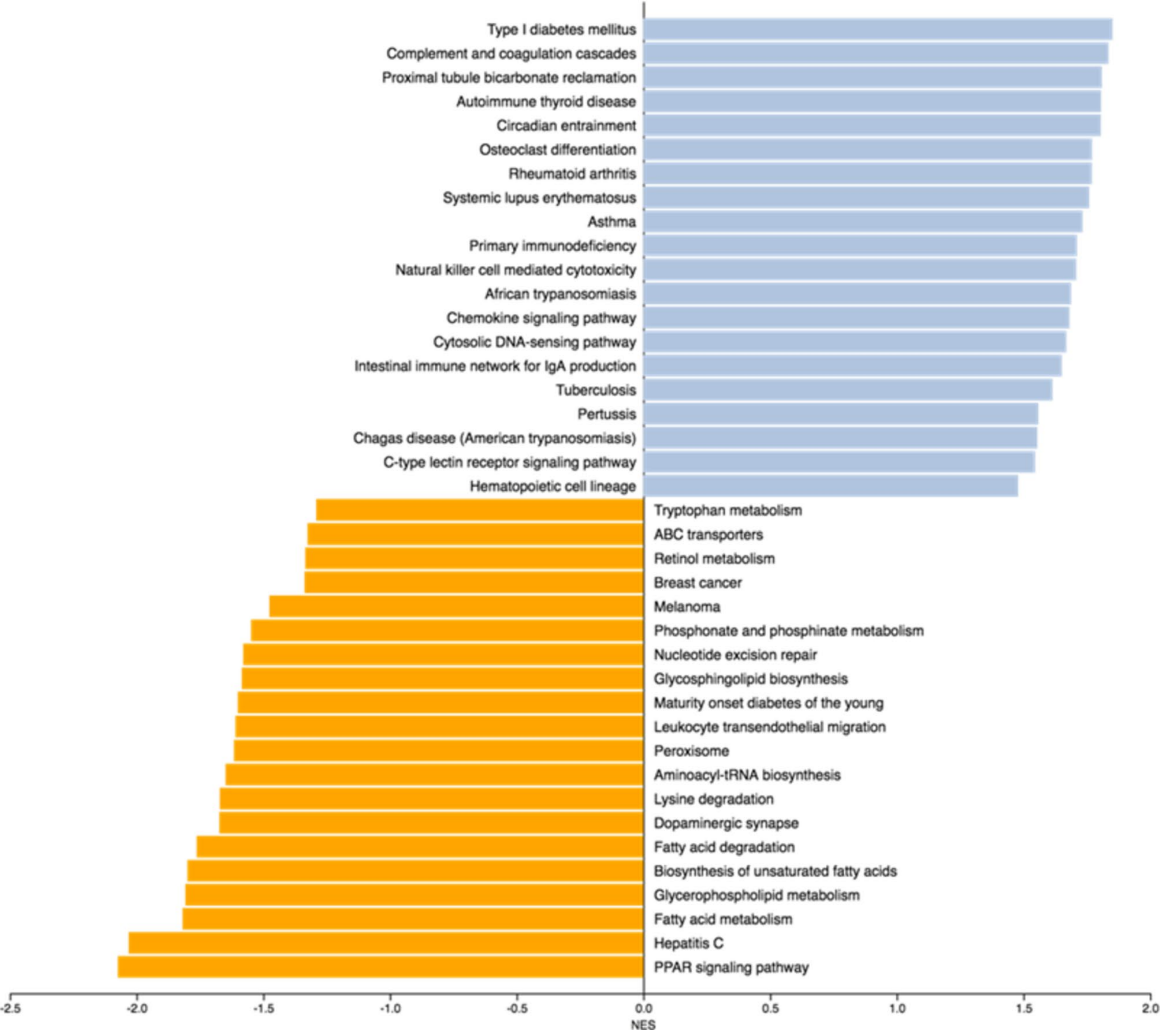
Pathways differentially enriched in GK rats following vertical sleeve gastrectomy (VSG) or sham operation. Gene set enrichment analysis (GSEA) of the liver transcriptomes of gastrectomized (n = 4) and sham operated (n = 4) GK rats 90 days post-surgery was used to identify KEGG pathways up- or down-regulated, as illustrated by measures of a normalized enrichment score (NES). Blue bars indicate positive enrichment scores and orange bars indicate negative enrichment scores. Details of statistics supporting pathway differential enrichment and the contributing genes are given in Supplementary Tables S3 and S4, respectively. Details of rno pathways can be found at www.genome.jp/kegg.

To identify biological functions affected by VSG and improved glucose homeostasis consecutive to VSG in the GK rat, we carried out gene set enrichment analysis (GSEA) of KEGG pathways^21,22^. Among the 12,494 genes in our transcriptome dataset that were mapped to a unique EntrezGeneID, 5170 could be retrieved in KEGG and used for GSEA. A total of 33 biological pathways were significantly differentially enriched between VSG rats and sham operated controls (Fig. 3). Normalised Enrichment Scores (NES) identified significantly up-regulated (n = 19) or down-regulated (n = 14) pathways (Supplementary Table S2). Details of genes contributing to the differential enrichment of these KEGG pathways in the rat models are given in Supplementary Table S3. The most significantly enriched pathways that differentiated VSG and sham operated GK rats were related to inflammatory and autoimmune processes, including “Type I diabetes mellitus” (NES = + 1.851, *p* = 0.004), “complement and coagulation cascades” (NES = + 1.836, *p* = 0.017), “primary immunodeficiency” (NES = + 1.711, *p* = 0.019), “natural killer cell mediated cytotoxicity” (NES = + 1.708, *p* = 0.019) and “chemokine signalling” (NES = + 1.681, *p* = 0.025) (Fig. 3, Supplementary Table S2). “Circadian entrainment” was among the top pathways significantly enriched in gastrectomized GK rats (NES = + 1.805, *p* = 0.021). In contrast, several metabolic pathways were significantly down-regulated in VSG GK rats, including “PPAR signalling” (NES = − 2.074, *p* = 0.004), “fatty acid metabolism” (NES = − 1.818, *p* = 0.016), “biosynthesis of unsaturated fatty acids” (NES = − 1.799, *p* = 0.013), “peroxisome” (NES = − 1.616, *p* = 0.044) and “lysine degradation” (NES = − 1.671, *p* = 0.033), which incorporates several histone methyltransferases significantly differentially expressed between VSG and sham operated GK rats (Fig. 3, Supplementary Tables 2 and 3).

### Elements of the “PPAR signalling” pathway are down-regulated by VSG in the GK rat

The KEGG pathway PPAR signalling (rno03320) showed the strongest evidence of down-regulation in VSG GK rats, which may account for altered regulation of pathways related to fatty acid metabolism in this model (Fig. 3). Expression of both core transcription factors in this pathway encoding the peroxisome proliferator activated receptor alpha (*Ppara*) and the retinoid X receptor gamma (*Rxrg*) was significantly decreased in VSG rats (Log2fold change (FC) = − 0.874, *p* = 3.58 × 10^–5^ for *Ppara* and Log2FC = − 0.505, *p* = 0.003 for *Rxrg*) (Table 1). This is expected to have strong repercussions on the transcription of genes contributing to ketogenesis and fatty acid metabolism, which was a pathway (rno01212) significantly down-regulated in VSG rats. Significant alteration of the expression of the genes encoding 3-hydroxy-3-methylglutaryl-CoA synthase 2 (*Hmgcs2*) (Log2FC = − 0.780, *p* = 6.4 × 10^–4^), Acetyl-CoA C-acetyltransferase (*Acat2l1*) (Log2FC = + 0.74, *p* = 7.6 × 10^–4^), 3-oxoacid CoA-transferase (*Oxct1*) (Log2FC = + 1.05, *p* = 1.6 × 10^–3^), and both 3-hydroxybutyrate dehydrogenase 1 (*Bdh1*) (Log2FC = − 0.87, *p* = 7.5 × 10^–5^) and 2 (*Bdh2*) (Log2FC = + 1.06, *p* = 4.6 × 10^–4^) (Supplementary Table S2) suggests reduced ketogenesis in VSG rats. Down-regulated expression of genes involved in fatty acid oxidation (*Cpt1b, Cpt2*, *Acadl*, *Acadm*, *Acox1*), cholesterol metabolism (*Cyp8b1*), translocation of long-chain fatty acids across the plasma membrane (*Slc27a4*), fatty acid elongation (*Slc27a5*) and arachidonic acid metabolism (*Acsl4*, *Cyp4a1 Cyp4a2*) (Table 1) are also likely direct causes of *Ppara* and *Rxrg* decreased expression in VSG rats. Consistent with changes in lipid metabolism pathways in the transcriptome of VSG rats, expression of three of the five perilipin genes was significant reduced (Table 1), including perilipin 2 (*Plin2*) which was massively down-regulated (Log2FC = − 3.27, *p* = 4.2 × 10^–194^).

**Table 1:** Genes contributing to down-regulated enrichment of the KEGG biological pathway “PPAR signalling” (rno03320) in gastrectomized GK rats. FC, fold change.

Acronym	Gene description	log2FC	Adjusted *p*
*Acadl*	Acyl-CoA dehydrogenase, long chain	− 0.319	0.020
*Acadm*	Acyl-CoA dehydrogenase, medium chain	− 0.624	0.024
*Acox1*	Acyl-CoA oxidase 1	− 0.456	0.014
*Acsl4*	Acyl-CoA synthetase long-chain family member 4	− 0.635	8.68 × 10^–4^
*Angptl4*	Angiopoietin-like 4	− 1.800	1.36 × 10^–11^
*Cpt1b*	Carnitine palmitoyltransferase 1B	− 1.607	1.64 × 10^–18^
*Cpt2*	Carnitine palmitoyltransferase 2	− 0.727	3.26 × 10^–4^
*Cyp4a1*	Cytochrome P450, family 4, subfamily a, polypeptide 1	− 0.812	0.005
*Cyp4a2*	Cytochrome P450, family 4, subfamily a, polypeptide 2	− 0.885	0.015
*Cyp8b1*	Cytochrome P450 family 8 subfamily B member 1	− 0.678	0.027
*Ehhadh*	Enoyl-CoA hydratase and 3-hydroxyacyl CoA dehydrogenase	− 1.317	4.61 × 10^–5^
*Gk*	Glycerol kinase	− 1.182	3.04 × 10^–26^
*Hmgcs2*	3-hydroxy-3-methylglutaryl-CoA synthase 2	− 0.780	6.43 × 10^–4^
*Me1*	Malic enzyme 1	− 1.075	2.78 × 10^–8^
*Plin2*	Perilipin 2	− 3.270	1.15 × 10^–194^
*Plin4*	Perilipin 4	− 1.379	0.005
*Plin5*	Perilipin 5	− 1.692	2.32 × 10^–11^
*Ppara*	Peroxisome proliferator activated receptor alpha	− 0.874	3.58 × 10^–5^
*Rxrg*	Retinoid X receptor gamma	− 0.505	0.003
*Slc27a4*	Solute carrier family 27 member 4	− 0.352	0.009
*Slc27a5*	Solute carrier family 27 member 5	− 0.445	0.012

Altered regulation of inflammation and lipid metabolism was also identified when GSEA was carried out with Wikipathways (www.wikipathways.org) (Supplementary Fig. S1) and when over representation analysis (ORA) was performed using the Wikipathways and the KEGG (Supplementary Table S4) and Reactome (Supplementary Table S5) databases. Interestingly, the latter pointed to overrepresentation of five pathways related to bile acid synthesis.

### Transcription regulation of key elements of the molecular clock is strongly altered by VSG

Analysis of transcriptome data through both GSEA and ORA of KEGG and Panther pathways consistently pointed to changes in the regulation of the molecular clock in VSG rats (Fig. 3**, **Supplementary Tables S2 and S4). Consistent with the significant enrichment of the KEGG pathway “circadian entrainment” (rno04713) through GSEA (NES = + 1.805, *p* = 0.021) (Fig. 3**, **Supplementary Table S2), the pathway “circadian clock system” in the Panther database (P00015) was found significantly differentially regulated in the rat groups through both GSEA (NES = − 0.9340, FDR = 1.0) (Supplementary Fig. S2) and ORA (*p* = 0.0036, FDR = 0.1404). ORA also indicated that the KEGG pathway “circadian rhythm” (rno04710) showed the strongest ratio of enrichment in the liver transcriptome of VSG rats (ratio: 3.02, *p* = 0.0018) (Supplementary Fig. S3, Supplementary Table S4).

The genes that contributed the most to enrichment of the KEGG pathway “circadian entrainment” were period circadian regulators *Per1* (Log2FC = + 1.046, *p* = 4.2 × 10^–8^), *Per2* (Log2FC = + 2.000,* p* = 1.9 × 10^–9^) and *Per3* (Log2FC = + 3.793, *p* = 3.0 × 10^–102^) (Table 2). The KEGG pathway “circadian rhythm” (rno04710), which was overrepresented in VSG rats, included these three genes and differentially expressed genes encoding the aryl hydrocarbon receptor nuclear translocator-like (*Arntl*/*Bmal1*) (Log2FC = − 4.391,* p* = 8.0 × 10^–105^), the neuronal PAS domain protein 2 (*Npas2*) (Log2FC = − 3.647, *p* = 4.7 × 10^–82^), the clock circadian regulator (*Clock*) (Log2FC = − 1.298, *p* = 3.3 × 10^–49^), the basic helix-loop-helix family, member e40 (*Dec1*, *Bhlhe40*) (Log2FC = + 0.932, *p* = 0.004) and member e41 (*Dec2*, *Bhlhe41*) (Log2FC = + 2.839, *p* = 2.4 × 10^–13^), the nuclear receptor subfamily 1, group D, member 1 (*Nr1d1, Rev-Erα*) (Log2FC = + 1.352, *p* = 3.3 × 10^–4^), cullin 1 (*Cul1*) (Log2FC = − 0.223, *p* = 0.021), the RAR related orphan receptor A (*Rora*) (Log2FC = − 0.684,* p* = 1.2 × 10^–3^), the S-phase kinase associated protein 1 (*Skp1*) (Log2FC = − 0.222, *p* = 0.041) and the cryptochrome circadian regulator 2 (*Cry2*) (Log2FC = + 1.294; *p* = 1.2 × 10^–7^) (Supplementary Table S1).

**Table 2:** Genes contributing to the enrichment of the KEGG biological pathway “circadian entrainment” in gastrectomized GK rats. FC, fold change.

Acronym	Gene description	Log2FC	Adjusted *p*
*Adcy7*	Adenylate cyclase 7	0.861	0.007
*Camk2b*	Calcium/calmodulin-dependent Protein kinase II beta	1.373	0.006
*Camk2d*	Calcium/calmodulin-dependent Protein kinase II delta	0.703	0.052
*Gnai1*	G protein subunit alpha i1	0.523	0.027
*Gnai2*	G protein subunit alpha i2	0.522	0.011
*Gnb2*	G protein subunit beta 2	0.246	0.063
*Gng2*	G protein subunit gamma 2	0.825	0.020
*Gngt2*	G protein subunit gamma transducin 2	1.057	0.003
*Grin2a*	Glutamate ionotropic receptor NMDA type subunit 2A	1.583	0.001
*Gucy1b2*	Guanylate cyclase 1 soluble subunit beta 2	0.505	0.002
*Itpr3*	Inositol 1,4,5-trisphosphate receptor, type 3	0.600	0.042
*Per1*	Period circadian regulator 1	1.046	4.2 × 10^–8^
*Per2*	Period circadian regulator 2	2.000	1.9 × 10^–9^
*Per3*	Period circadian regulator 3	3.793	3.0 × 10^–102^
*Plcb4*	Phospholipase C, beta 4	0.437	0.034
*Prkcb*	Protein kinase C, beta	1.040	0.017
*Rps6ka5*	Ribosomal protein S6 kinase A5	0.603	0.021

**Fig. 4:**
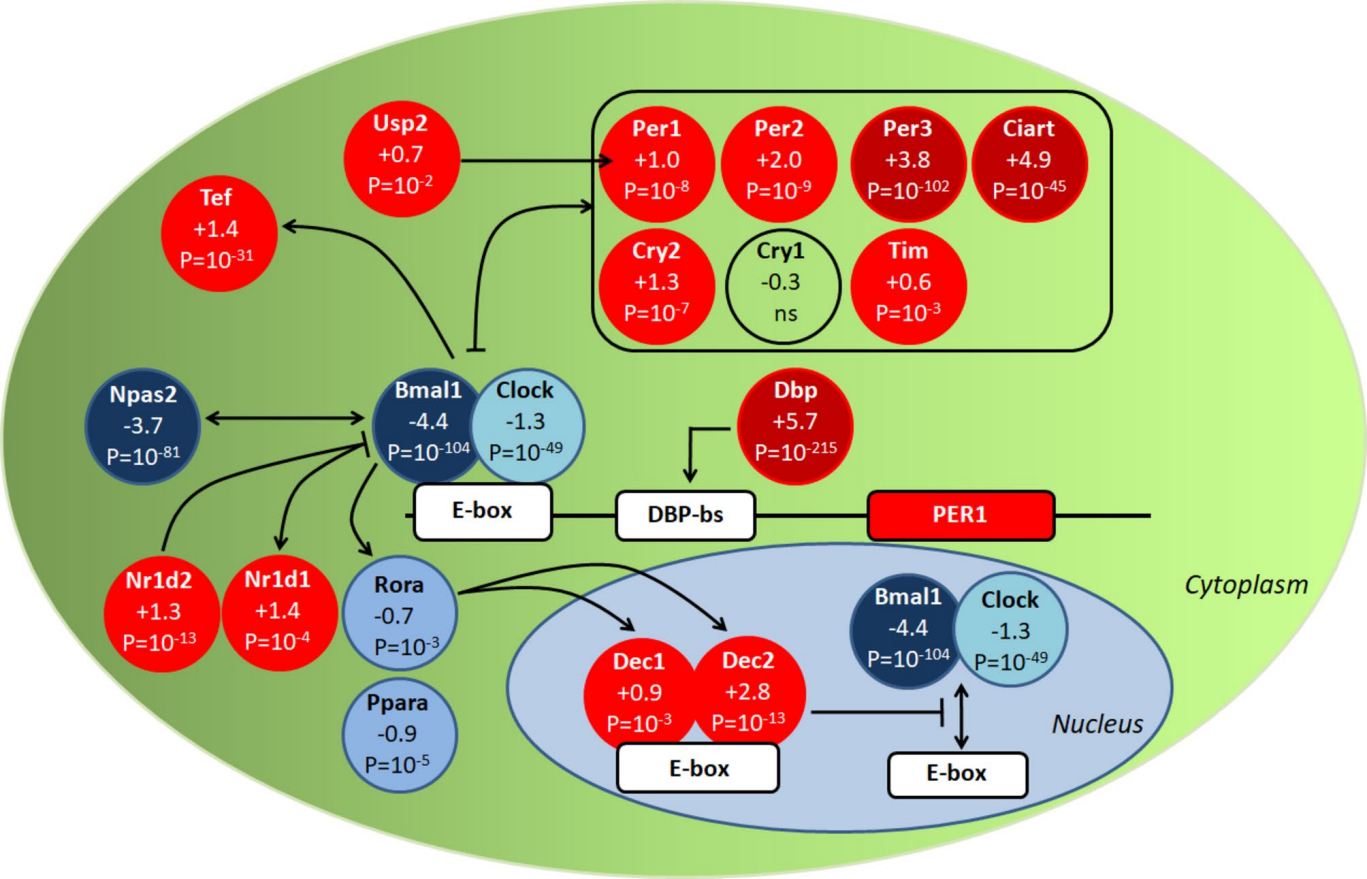
Schematic representation of liver transcription regulation of elements of the molecular clock in gastrectomized Goto-Kakizaki (GK) rats. Upregulated genes in gastrectomized rats are shown in red circles and down-regulated genes are shown in blue circles. Transcriptome data were generated using samples from four VSG rats and four sham-operated rats 90 days post-surgery. Log2 Fold changes and p-values corrected for multiple testing using the Benjamini and Hochberg method (see methods) are shown for each gene. Details of the genes are given in Supplementary Table S1.

Deeper analyses identified altered expression of other elements of the molecular clock in VSG rats, including the D-box binding PAR bZIP transcription factor (*Dbp*), which was massively upregulated following VSG (Log2FC = + 5.765, *p* = 3.8 × 10^–216^), the circadian associated repressor of transcription (*Ciart*) (CHRONO) (Log2FC = + 4.893,* p* = 8.9 × 10^–46^), the nuclear receptor subfamily 1, group D, member 2 (*Nr1d2, Rev-Erβ*) (Log2FC = + 1.291,* p* = 8.0 × 10^–14^), the TEF transcription factor, PAR bZIP family member (*Tef*) (Log2FC = + 1.382,* p* = 5.7 × 10^–32^), timeless (*Tim*) (Log2FC = + 0.644,* p* = 0.004) and the ubiquitin specific peptidase 2 (*Usp2*) (Log2FC = + 0.740, *p* = 0.038) (Fig. 4, Supplementary Table S1). Strikingly, 6 of the top 12 genes showing the strongest evidence of statistically significant differential expression in the transcriptome of VSG rats are central components of the molecular clock (*Dbp*, *Arntl*, *Per2*, *Npas2*, *Clock*, *Ciart*). Transcription of downstream targets of clock genes (eg. *Insig2*, *Elovl5*, *Acox1*, *Hadh*, *Hadha*, *Hadhb*, *Hnf4a*, *Hnf4g*, *Stat5a*, *Stat5b*) was down-regulated in VSG GK rats. These results indicate that coordinated and biologically coherent alteration in the expression of almost all known components of the hepatic molecular clock is a hallmark of the adaptation to VSG in a model of spontaneously-occurring lean T2D.

We confirmed by quantitative RT-PCR statistically significant differential expression of genes involved in inflammatory processes and the molecular clock (*Il1β*, *Il10*, *Ccl2*, *Arntl*, *Ciart*, *Cry2*, *Clock*, *Npas2*, *Per1*, *Per2*, *Per3* and *Rorα*) between VSG and sham operated GK rats (Supplementary Fig. S4).

### VSG results in altered hepatic expression of genes involved in epigenome regulation

We have previously reported in the GK strain alterations in the genetic and hepatic transcriptional regulation of protein acetylation^23^, a post-translational mechanism under circadian clock control and involved in the expression of core clock genes and clock-controlled genes^24^. Several genes encoding canonical and non-canonical acetyltransferases and deacetylases, or involved in protein acetylation, were found significantly downregulated (*Acat1*, *Atxn1*, *Atxn3*, *Crat*, *Gnpat*, *Hdac5*, *Kat2a*, *Kat8*, *Kat14*, *Lpcat3*, *Ncor1*, *Pafah1b1*) or upregulated (*Amdhd2*, *Hdac1*, *Hdac7*, *Hdac11*) following VSG in GK rats (Supplementary Table S1). We also noted significant downregulation of several lysine demethylases (*Kdm1a*, *Kdm2a*, *Kdm2b*, *Kdm3a*, *Kdm7a*) and histone methyltransferases (*Dot1l*, *Kmt2a*, *Kmt2d*, *Kmt5b*, *Setdb2*), and upregulation of the lysine methyltransferases *Kmt5c* and *Suv39h1* in the liver of VSG GK rats (Table 3**, **Supplementary Table S1). These findings suggest that epigenetic mechanisms in the liver occur in diabetes remission and the response to bariatric surgery.

**Table 3:** Genes encoding histone methyltransferases that contribute to the enrichment of the KEGG biological pathway “Lysine degradation” in gastrectomized GK rats. FC, fold change.

Acronym	Gene description	log2FC	Adjusted *p*
*Acat1*	acetyl-CoA acetyltransferase 1	− 0.651	0.031
*Aldh2*	aldehyde dehydrogenase 2 family member	− 0.297	0.024
*Bbox1*	gamma-butyrobetaine hydroxylase 1	− 0.680	0.006
*Dot1l*	DOT1 like histone lysine methyltransferase	− 0.660	3.56 × 10^–6^
*Ehhadh*	Enoyl-CoA hydratase and 3-hydroxyacyl CoA dehydrogenase	− 1.317	4.61 × 10^–5^
*Ehmt1*	euchromatic histone lysine methyltransferase 1	− 0.345	0.076
*Hadh*	hydroxyacyl-CoA dehydrogenase	− 0.586	0.044
*Hadha*	hydroxyacyl-CoA dehydrogenase trifunctional multienzyme complex subunit alpha	− 0.467	0.003
*Kmt2a*	lysine methyltransferase 2A	− 0.498	0.004
*Kmt2c*	lysine methyltransferase 2C	− 0.473	0.069
*Kmt2d*	lysine methyltransferase 2D	− 0.347	1.40 × 10^–5^
*Kmt5a*	lysine methyltransferase 5A	− 0.297	0.149
*Kmt5b*	lysine methyltransferase 5B	− 0.234	0.015
*Nsd1*	nuclear receptor binding SET domain protein 1	− 0.351	0.033
*Nsd2*	nuclear receptor binding SET domain protein 2	− 0.410	0.007
*Nsd3*	nuclear receptor binding SET domain protein 3	− 0.256	0.123
*Pipox*	pipecolic acid and sarcosine oxidase	− 0.633	0.067
*Plod1*	procollagen-lysine, 2-oxoglutarate 5-dioxygenase 1	− 0.297	0.026
*Prdm2*	PR/SET domain 2	− 0.232	0.165
*Setd1b*	SET domain containing 1B, histone lysine methyltransferase	− 0.351	0.083
*Setd2*	SET domain containing 2, histone lysine methyltransferase	− 0.176	0.170
*Setdb1*	SET domain bifurcated histone lysine methyltransferase 1	− 0.193	0.185
*Setdb2*	SET domain bifurcated histone lysine methyltransferase 2	− 0.652	3.51 × 10^–5^

### Liver metabolome and lipidome profiling suggest downregulation of lysophosphatidylethanolamine and phosphatidylcholine metabolism by VSG

**Fig. 5:**
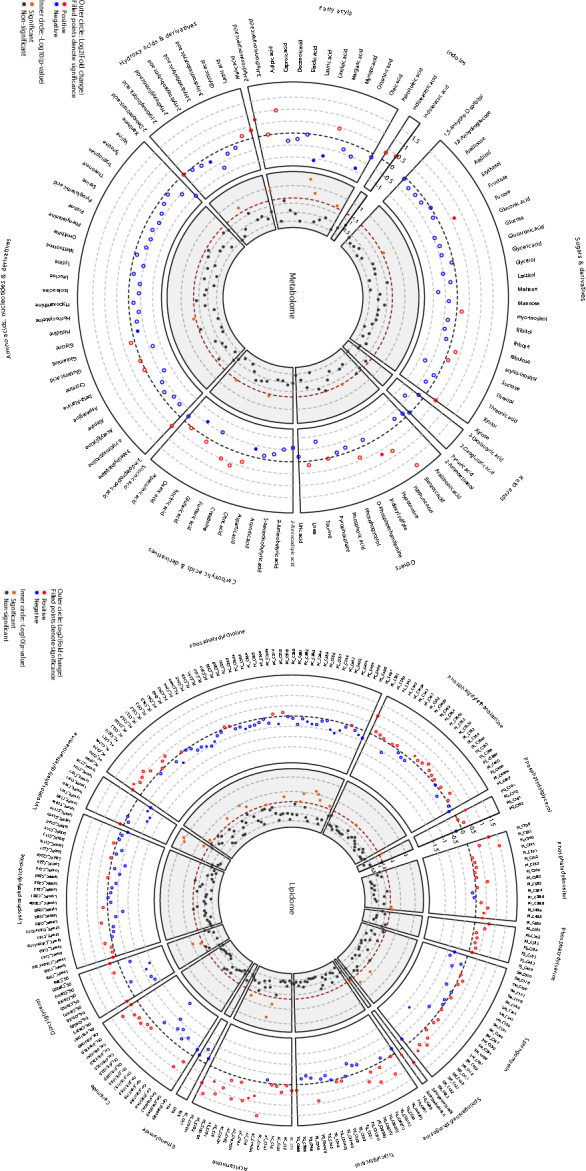
Alterations in liver metabolome and lipidome profiles in gastrectomized GK rats. GC- and LC–MS were used to derive semi-quantitative data for small molecular weight metabolites (left) and lipids (right), respectively, in liver of VSG (n = 4) and sham-operated (n = 4) GK rats 90 days post-surgery. The inner circle displays the -log10(p-value) of quantitative changes. Significant and non-significant changes in the level of metabolites and lipids between VSG and sham-operated GK rats are shown in orange and black dots, respectively. Dots in the outer circle indicate increased (red dots) or decreased (blue dots) levels of the metabolites and lipids in VSG GK rats. Details of metabolites and lipids that were detected by our MS methods and used in the analyses are given in Supplementary Tables S6 (metabolome) and **S7** (lipidome).

Elevated hepatic triglycerides and anomalies in the regulation of liver lipid metabolism spontaneously occur in the GK rat^25^. To identify biological processes that may explain improved glycemic control and liver transcriptional changes of PPAR signalling and the metabolism of fatty acids in VSG rats, we carried out metabolome and lipidome profiling in liver samples of gastrectomized and sham operated rats. Metabolome and lipidome analytical methods were designed to semi-quantify a total of 114 metabolites (Supplementary Table S6) and 380 lipids (Supplementary Table S7), respectively. Eight metabolites were not detected in liver extracts (citrulline, cysteinylglycine, uridine, 2-Oxoisocaproic acid, 3-Methyl-2-oxobutyric acid, 3-Methyl-2-oxovaleric acid, 4-Hydroxybenzoic acid and 4-cresol) (Supplementary Table S6). We identified significant reduction in liver levels of aspartic acid (Log2FC = − 0.35, *p* = 0.039), glycine (Log2FC = − 0.23, *p* = 0.038), lauric acid (Log2FC = − 1.08, *p* = 0.004), linoleic acid (Log2FC = − 0.82, *p* = 0.020), oleic acid (Log2FC = − 0.87, *p* = 0.038), oxalic acid (Log2FC = − 0.57, *p* = 0.045) and 2-hydroxyisobutyric acid (Log2FC = − 0.24, *p* = 0.025) in VSG GK rats when compared to sham operated GK rats (Fig. 5**, **Supplementary Table S6). Liver levels of fucose (Log2FC = 0.65, *p* = 0.041), hypotaurine (Log2FC = 0.68, *p* = 0.047) and 2-hydroxyisovaleric acid (Log2FC = 0.73, *p* = 0.015) were increased in VSG rats.

Among the eighteen lipid classes that could be analyzed with our LC–MS method, cholesteryl esters (n = 32), dihexosylceramides (n = 6), gangliosides (n = 6), glucosylceramides (n = 19) and trihexosylceramides (n = 6) were largely or completely undetected in rat liver extracts, and only 42% (10/21) of the diacylglycerols and 53% (25/47) of the triacylglycerols were analysed (Supplementary Table S7). Among lipids that could be detected in liver extracts, there were no significant differences in the levels of ceramides, ethanolamides, lysophosphatidylcholines, phosphatidylserines and triacylglycerols between VSG and sham-operated GK rats (Fig. 5**, **Supplementary Table S7). The sphingosine (d18:1) and only few lipids of the acylcarnitine (AC(16:0), AC(16:1), AC(18:2)), diacylglycerol (DG(36:4)), sphingomyelin (SM(40:2), SM(42:2)), phosphatidylinositol (PI(40:5), PI(40:6)), phosphatidylglycerol (PG(34:1)) and phosphatidylethanolamine (PE(36:4)) classes showed significantly different hepatic levels between VSG and sham-operated GK rats. Lipid classes that were mostly affected by gastrectomy were phosphatidylcholines (PC) and lysophosphatidylethanolamines (LPE), with significantly decreased liver amounts of PC(33:3) (Log2FC = − 0.44, *p* = 0.020), PC(34:4e) (Log2FC = − 0.41, *p* = 0.011), PC(35:0) (Log2FC = − 0.38, *p* = 0.022), PC(36:2) (Log2FC = − 0.33, *p* = 0.049), PC(37:4) (Log2FC = − 0.36, *p* = 0.022), PC(38:4) (Log2FC = − 0.34, *p* = 0.013), PC(39:6) (Log2FC = − 0.46, *p* = 0.006), PC(40:1) (Log2FC = − 0.58, *p* = 0.009), PC(40:2) (Log2FC = − 0.38, *p* = 0.021), PC(40:6) (Log2FC = − 0.29, *p* = 0.031), LPE(18:1) (Log2FC = − 0.95, *p* = 0.001), LPE(18:2) (Log2FC = − 0.98, *p* = 0.001) and LPE(20:4) (Log2FC = − 0.73, *p* = 0.024) in VSG rats when compared to sham operated rats (Fig. 5**, **Supplementary Table S7).

These results provide information on metabolites and lipids that are present in the liver or can be detected by our MS method, and indicate that VSG affects predominantly lipids of the phosphatidylcholine and lysophosphatidylethanolamine classes.

### Gene expression patterns suggest therapeutic consequences of VSG

**Fig. 6:**
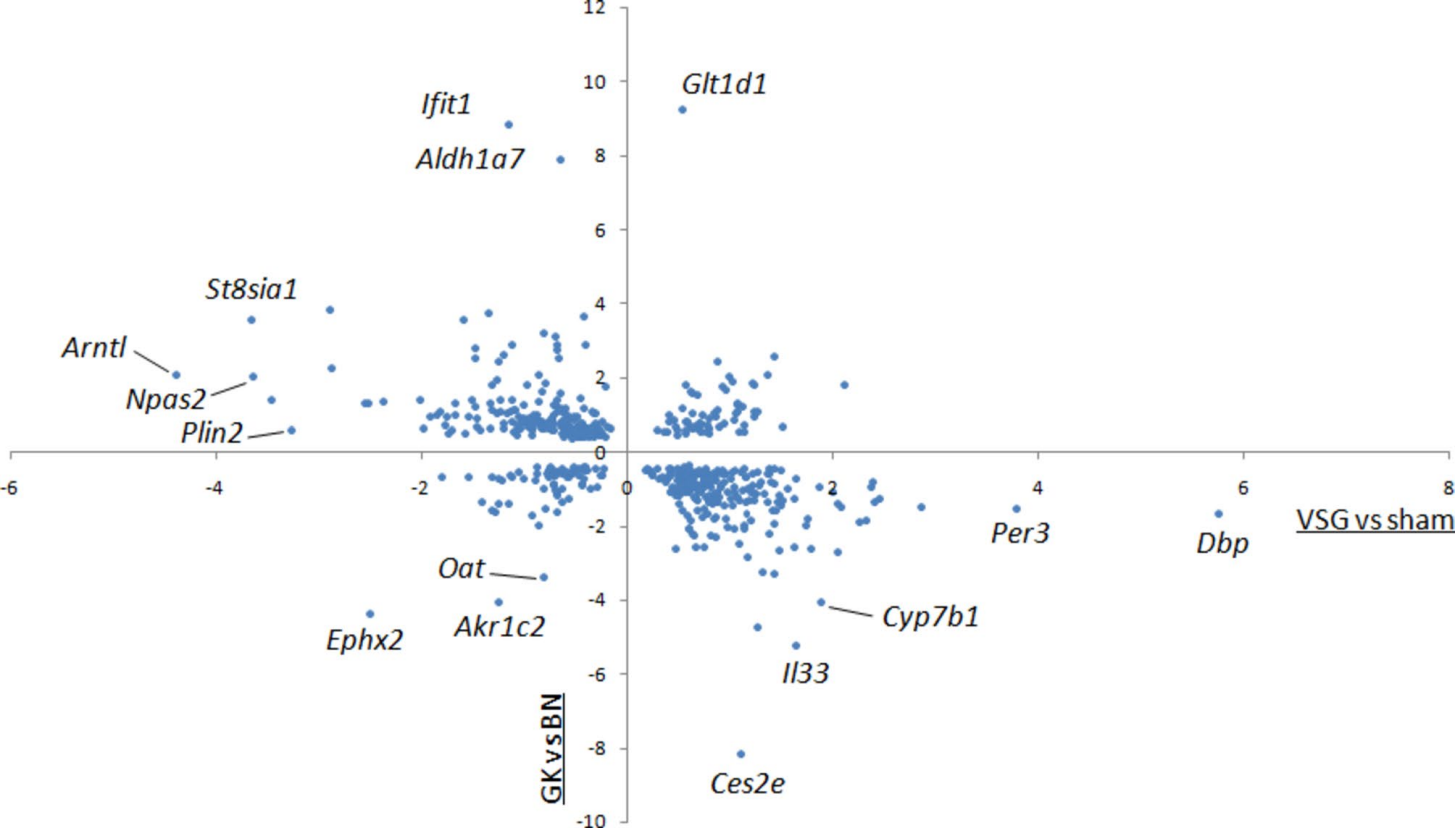
Comparative analysis of differentially expressed genes between gastrectomized (VSG) Goto-Kakizaki (GK) rats and sham-operated GK rats and between GK and normoglycemic rats of the Brown Norway (BN) strain. Normalised enrichment scores (NES) of the 567 genes significantly differentially expressed in liver in the two comparisons are plotted to illustrate divergent and conserved gene expression patterns. Transcriptome (RNA sequencing) data in GK and BN rats are from Kaisaki et al.^23^. Details of the statistics and gene description are given in Supplementary Table S8.

To disentangle the possible pathophysiological relevance of VSG-promoted changes in liver gene expression and consecutive improvement in glucose homeostasis, we compared results from liver RNA sequencing in VSG and sham operated GK rats to those previously generated in GK/Ox and normoglycemic inbred rats of the Brown Norway (BN) strain^23^. Rats in the two studies are age-matched males and were maintained in identical conditions and were killed in the same time window for organ collection. Despite marked genetic differences between the inbred GK and BN strains^26^, among the 3102 genes differentially expressed between gastrectomized GK and sham operated GK, only 567 (18%) were also differentially expressed between GK and BN (Supplementary Table S8). Of note, as many as 437 of these genes (77%) showed opposite expression patterns (Fig. 6). Contrasting expression patterns in the two comparisons was particularly remarkable for several genes involved in the molecular clock (*Arntl/Bmal1*, *Dbp*, *Npas2*, *Per1*, *Per2*, *Per3*, *Tef*, *Tim* and *Usp2*) (Supplementary Fig. S5, Supplementary Table S8). The remaining genes of the molecular clock differentially expressed between gastrectomized GK and sham operated GK did not show evidence of significant differential expression between GK and BN rats. In spite of genetic differences between GK and BN that may account for the contrasting transcriptional control of clock genes in the two comparisons, in the absence of a proper normoglycemic control produced specifically for the GK strain, these findings indicate that the regulation of the hepatic clock is conserved in models of normoglycemia and experimentally-induced diabetes remission.

Collectively, the profound remodeling of hepatic gene expression and evidence of alternative wiring of the liver molecular clock in response to VSG may contribute to the therapeutic consequences of bariatric surgery in lean rats of the GK strain through nycthemeral alterations in feeding patterns and activity, and underline the impact of chronobiology in etiopathogenesis of non-obese T2D.

## Discussion

We report results from metabolic, behavioral and molecular adaptations to bariatric surgery in the GK rat model of non-obese type 2 diabetes (T2D), which suggest the involvement of chronobiology and the epigenome in T2D remission. We show that VSG in the GK strain promotes changes in nycthemeral feeding patterns and activity and in hepatic molecular mechanisms linked to the circadian clock, inflammation, lipid metabolism and histone modification, which complement previously documented alterations in the gut microbiota and the metabolism of bile acids in this model of gastrectomy-induced diabetes remission^14^. These results underline the central role of the liver in the complex regulation of multiple biological mechanisms that collectively contribute to surgically-induced improvement in glucose homeostasis in a model characterized by spontaneously-occurring glucose intolerance in the absence of confounding effects of obesity.

We provide confirmatory evidence of the beneficial effects of bariatric surgery on glycemic control, which we, and others, have previously demonstrated in the GK strain^12–14,27–30^ and in preclinical models of obesity and T2D either induced by high fat diet (HFD) feeding^31^ or genetically engineered^32,33^. The lean T2D GK strain used in this study allows investigations of the therapeutic consequences of bariatric surgery in the absence of the confounding effects of weight loss. As reported in lean C57BL6/J mice^34^, GK rats exhibit temporary reduction in body weight immediately following VSG, gastrectomized rats returning within five weeks to body weight of their lean littermates. This delay in body weight recovery may be explained by sustained reduction in food intake following VSG and maintenance on a carbohydrate-rich diet. In contrast to previous studies using diet-induced obese or Wistar rats, where food intake typically normalizes within weeks after VSG^18,35^, total food intake remained lower in GK rats 3 months after surgery. This sustained reduction may be related to the intrinsic pathophysiological features of the GK strain, combining impaired glucose sensing, altered gut–brain signalling, and reduced compensatory hyperphagia^36,37^, which could potentiate the long-term anorexigenic effects of VSG and partly explain the persistent gap in cumulative food intake observed in our study. The observation of similar body weight in VSG and sham-operated GK rats despite lower food intake in VSG rats may be explained by the combination of a reduction in caloric intake in the VSG group and a proportional decrease in energy expenditure, likely reflecting a compensatory adjustment in metabolic rate. This balance between energy intake and expenditure highlights the importance of considering both sides of the energy balance equation when interpreting post-surgical weight trajectories. Improved glycemic control in VSG GK rats is therefore the likely direct consequence of bariatric surgery and involves biological mechanisms independent to weight loss.

Transcriptome data in VSG GK rats suggest a decrease in lipid-related pathways and reduction of fatty liver through down-regulated expression of both *Plin2* and *Plin5*. This is supported by reports of protection against fatty liver disease following inactivation of *Plin2*^38^ and *Plin5*^39^. Inhibited expression of *Plin2* and changes in bile acid synthesis in gastrectomized GK rats in our study support reports of improved fatty liver disease^40^ and increased bile acid metabolism^41^ following bariatric surgery. Downregulated hepatic expression on *Plin2* was also reported in obese patients following Roux-en-Y gastric bypass^42^. Further evidence of altered lipid regulation in gastrectomized GK rats is illustrated by decreased liver levels of fatty acyls (lauric acid, oleic acid, linoleic acid) and a pattern of hepatic depletion of diacylglycerols (DAG), phosphatidylcholines (PC), lysophosphatidylcholines (LPC) and lysophosphatidylethanolamines (LPE), and increased levels of acylcarnitines (AC), phosphatidylethanolamines (PE), phosphatidylinositols (PI) and phosphatidylserines (PS). Counterintuitive increased expression of the stearoyl-Coenzyme A desaturase *Scd2* and members of the phospholipase A2 family (*Pla2g2a*, *Pla2g2d*, *Pla2g7*, *Pla2g16*), which produces LPE from PE, in VSG GK rats suggests the involvement of cellular mechanisms stimulating LPE export. Interestingly, results from metabolomic analyses have shown that LPE(20:4) and LPE(18:1), which are differentially regulated in VSG GK rats in our study, are induced by PPARα activation in hepatocytes^43^ and that PC species may be an endogenous PPARα ligand^44^. In addition, LPE 18:2, which is also significantly decreased in VSG rats, exhibits adverse effects on liver function by suppressing lipolysis and fatty acid biosynthesis in a human liver cell line^45^. Increased levels of hepatic AC(16:0), AC(16:1) and AC(18:2) in VSG GK rats suggest accumulation of long-chain acylcarnitines and decreased fatty acid beta-oxidation capacity, which may be linked to the downregulation of the PPAR pathway.

Activation of the farnesoid X receptor (FXR, NR1H4) and down-regulated expression of cholesterol 7α-hydroxylase (CYP7A1) account for the beneficial effects of bariatric surgery on glucose homeostasis^46,47^. However, down-regulated expression of *Fxr* (Log2FC = − 0.378, *p* = 0.016) and upregulated expression of *Cyp7a1* (Log2FC = + 1.440, *p* = 1.2 × 10^–5^) in liver of VSG GK rats in our study agree with improved hepatic steatosis and insulin sensitivity and increased bile acid metabolism in mice overexpressing *Cyp7a1*^48^. These mice also exhibit decreased expression of *Cyp8b1*, which we found downregulated in VSG GK rats (Log2FC = − 0.678, *p* = 0.027), and which contribute to increase the pool of bile acids as demonstrated in *Cyp8b1* knockout mice^49^. These data suggest that stimulation of bile acid synthesis may occur in VSG GK rats through coordinated changes in the expression of *Cyp7a1* and *Cyp8b1*.

Recording of feeding and activity in gastrectomized and sham operated GK rats suggests that VSG results in modulating chronobiology and chrono-nutrition. Gastrectomized GK rats exhibited fragmented meal patterns during both the light and dark phases resulting in increased food intake during the light phase, which is a resting period in rodents, and reduced food intake during the dark phase, when rodents normally feed, despite counterintuitive increased activity during the latter phase. Overall food intake was reduced in VSG GK rats when compared to sham operated GK controls. Relationships between bariatric surgery and meal frequency have been addressed in obese mice^50,51^. Obese patients who underwent bariatric surgery exhibit changes in time-of-day regulation in liver gene transcription^52^. However, the direct involvement of the circadian clock in rhythmic food intake through expression of core clock genes remains debated^53,54^. The anatomical and functional changes induced by bariatric surgery in GK rats lead to smaller, more frequent meals throughout the light–dark cycle, likely caused by a reduction in stomach volume, which may alter nutrient-driven entrainment of the liver molecular clock. Temporal redistribution of feeding could re-align hepatic circadian rhythms, coordinating gluconeogenesis, glycogen storage, and lipid metabolism to improve glucose tolerance. These chronobiological mechanisms provide a plausible link between altered meal timing and the metabolic benefits of VSG in GK rats in our study, although single-time-point sampling precludes direct assessment of circadian rhythms.

Coordinated changes in the expression of many genes involved in the hepatic molecular clock are one of the most remarkable transcriptional consequences of VSG in GK rats, which may result in altered feeding patterns and activity in these rats. Core clock genes synchronise transcriptional and translational feedback loops and regulate metabolism in health and disease^55,56^. Liver transcriptome studies in experimental systems of T2D and obesity have consistently documented changes in the expression of genes involved in the circadian regulation^57^. Conversely, RYGB in obese mice fed high fat diet results in changes in hepatic expression of *Clock*, *Bmal1*, *Per1* and *Per2*^51^. Diabetes remission following VSG in GK rats is associated with increased expression of *Dbp*, *Per1*, *Per2* and *Per3* and decreased expression of *Naps2*, *Arntl/Bmal1* and *Clock*, which fit the known feedback loop transcriptional regulation of these genes in the pathway. We have shown that none of these genes were significantly differentially expressed in brain regions (hypothalamus, hippocampus, striatum, brainstem) between VSG GK rats and sham controls^15^ indicating an exclusive transcriptional effect of VSG on the molecular clock in the liver rather than in the central nervous system. The relevance of hepatic expression patterns of these genes to T2D remission in humans is supported by the identification of altered regulation of pathways related to the circadian clock in islets from individuals with early-stage T2D, and potentially T2D-causing mechanisms, characterized mainly by increased expression of *Clock* and decreased expression of *Per1*, *Per2*, *Ciart*, *Cry2* and *Usp2*^58^. Expression on *Per1* and *Cry1* was also found downregulated in liver of obese patients following Roux-en-Y gastric bypass^42^. Downregulated hepatic expression of genes involved in hepatic fatty acid oxidation and PPAR signalling in VSG GK rats and above mentioned reduced liver levels of linoleic acid, a ligand and activator of PPARα^59^ which influences the liver molecular clock^60^, may be central in transcriptional adaptation of the molecular clock to gastrectomy and in improved glucose tolerance. It may reflect a metabolic shift that reduces hepatic gluconeogenesis from fatty acid-derived acetyl-CoA and promotes peripheral substrate utilization, consistent with reports suggesting that reduced hepatic fatty acid oxidation can paradoxically enhance systemic glucose homeostasis.

The strongest and most significantly upregulated gene in response to VSG in the GK rat was *Dbp*, which is proposed as an upstream regulator of the clock system^61^. Of note, genes regulating nicotinamide metabolism (*Nampt* and *Nmrk1*), which are rhythmically expressed in liver and involved in the autonomous hepatic clock^62^, were upregulated in VSG rats. Strongly discordant expression patterns of central elements of the molecular clock (*Arntl/Bmal1*, *Dbp*, *Npas1*, *Per1*, *Per2*, *Per3*, *Tef*, *Usp2*) between VSG GK rats and sham-operated GK controls, and between GK and normoglycemic BN rats^23^, suggests that the therapeutic contribution of VSG in GK rats involves alternative wiring of the liver clock transcriptome.

Our liver transcriptome data suggest that diabetes remission and alteration of the liver molecular clock following VSG involve epigenetic mechanisms, which we have documented in the GK strain^23^, through changes in the hepatic expression of several genes encoding acetyltransferases, deacetylases and lysine demethylases and methyltransferases (e.g. *Hdac1*, *Hdac5*, *Hdac7*, *Hdac11*, *Kdm1a*, *Kdm2a*, *Kdm2b*, *Kdm3a*, *Kdm7a*, *Kmt2a*, *Kmt2d*, *Kmt5b*, *Setdb2*). The role of histone methylation in metabolic regulation and chronic diseases has been extensively documented^63^, and there is increasing evidence that epigenetic mechanisms occur in response to weight loss and improved glucose homeostasis following bariatric surgery^64,65^. Histone lysine acetylation is a post-translational modification system, which displays rhythmic oscillation in liver and is critical for the transcription of core clock genes and clock-controlled genes^24^. CLOCK itself exhibits intrinsic lysine acetyltransferase properties and may mediate rhythmic acetylation of protein substrates^24^. Increased expression of *Sorbs3* (*p* = 5.5 × 10^–18^), *Ptpre* (*p* = 0.006) and decreased expression of *Pdk4* (*p* = 0.048) and *Dnmt3b* (*p* = 1.2 × 10^–11^) in VSG GK rats further support the involvement of epigenetic mechanisms in diabetes remission and in the long-term adaptation to bariatric surgery. Decreased promoter methylation and increased transcription of SORBS3 have been reported in obese patients following weight loss consecutive to bariatric surgery^66^. In contrast, post-bariatric surgery hypermethylation and transcription downregulation were identified for PDK4 in obese patients^67^ and PTPRE in patients with liver steatosis^68^. In addition, the lysine methyltransferase *Suv39h1*, which we found upregulated in gastrectomized GK rats, has been shown to repress the transcriptional activity of CLOCK-BMAL1^69^. Downregulation of the pathway aminoacyl-tRNA biosynthesis in VSG rats, which is the most affected pathway by bariatric surgery^70^, further supports a role of translation and post-translational regulations in gastrectomy-promoted diabetes remission.

## Conclusions

Our findings underline the breadth of behavioural and hepatic molecular mechanisms that collectively contribute to sustained improvement of glycemic control consecutive to bariatric surgery in a genetic model of T2D devoid of obesity. Our findings suggest that VSG improves glucose homeostasis through a combination of altered meal timing, redistribution of energy substrates and hepatic metabolic remodeling. Altered hepatic expression of *Pparα* and genes involved in the regulation of histone methylation and acetylation, including CLOCK, and aminoacyl-tRNA biosynthesis in VSG GK rats suggests a priming role of translational and post-translational mechanisms in the control of the circadian clock and in gastrectomy-promoted diabetes remission in GK rats. This is further supported by reports showing that inhibition of the lysine demethylase *Kdm1a* reduces energy expenditure^71^ and improves glycemic control and reduces obesity^72^. In addition, evidence of regulation of *Cyp7a1* and *Insig2* by *Nr1d1*^73^, binding of RORA and NR1D1 to the promoter of *Cyp8b1* to stimulate histone acetylation and control rhythmic expression^74^, and binding of DBP to the promoters of *Cyp7a1*^75^ and *Per2*^76^, strongly suggest connections between bile acid metabolism, histone modification and the circadian clock in VSG GK rats. Our findings provide enhanced knowledge of the central role of the liver in the regulation of biological mechanisms associated with diabetes remission following bariatric surgery, and important insights for the development of non-surgical strategies for the treatment of T2D and obesity.

### Limitations of the study

Causal relationships between physiological improvements in response to bariatric surgery and changes in nycthemeral feeding patterns and activity, in the regulation of metabolome and lipidome features, in the transcription of genes involved in the liver molecular clock and in histone modification, as well as in gut microbiota architecture and bile acid metabolism that we previously identified in gastrectomized GK rats^14^, remain to be disentangled through deeper analyses at multiple time points in order to assess the impact of circadian rhythms on these phenomena. We provide information on extreme steps of genome expression, from transcripts to metabolites and lipids, affected by VSG and improved glycemic control, but protein functions may also be involved. Our previous report of significant changes in the brain transcriptome, mostly related to the hypothalamus endothelium and components of the extra cellular matrix, in VSG GK rats^15^, suggests that other organs than liver contribute to VSG-promoted diabetes remission.

## Methods

### Animals

Inbred Goto-Kakizaki (GK/Ox) rats from a colony maintained at CNRS UMR 8251 were bred in individually ventilated cages. Rats were bred in controlled conditions of maintenance of 12 h dark–light cycles, temperature (22–24 °C) and relative humidity (50–60%). They had access to water and standard chow (SAFE, Augy, France) ad libitum. All procedures were carried out in male rats. All animal experimental protocols were approved by the Charles Darwin Ethics Committee in Animal Experiment at the University Paris Cité, Paris, France. They were carried out in accordance national guidelines and regulations under national licence condition (Ref. 5611 2,016,060,311,046,952 v2) following ethical review. All methods are reported in accordance with ARRIVE guidelines.

### Bariatric surgery in GK rats and post-operative monitoring

Following results from a previous study that documented the model of vertical sleeve gastrectomy (VSG) in GK rats in our experimental conditions^14^, VSG was performed using a new batch of GK rats. Briefly, 14-week-old male GK rats were anesthetized and the lateral 80% of the stomach was excised to leave a tubular gastric remnant in continuity with the oesophagus, the pylorus and the duodenum. Control GK rats were sham operated through application of pressure with forceps along the oesophageal sphincter and the pylorus. Blood glucose was monitored at weekly interval before the operation and over a period of 84 days afterwards using an Accu-Check® Performa glucometer (Roche Diagnostics, Meylan, France). Body weight and food intake were measured daily 30 days before the operation and until the end of the experiment. Fat mass and lean tissue mass were recorded in VSG and sham operated GK rats on the day of surgery and 26, 33, 52, 76 and 90 days afterwards using an Echo Medical Systems Echo MRI 100 (Whole Body Composition Analysers, EchoMRI, Houston, USA).

### Nycthemeral feeding pattern recording and activity analysis

Three months after bariatric surgery, when body weight of VSG-treated GK rats returned to values of sham operated GK controls, spontaneous feeding and locomotor activity were measured using an automated system of feeding and activity recording (Labmaster, TSE Systems, Bad Homburg, Germany). Food consumption was recorded every 160 min over a period of three days. Diurnal and nocturnal activities were recorded using an infrared light beam-based locomotion monitoring system (beam breaks/h).

### Sample collection

At the end of the experiment (90 days post-surgery), rats were fasted overnight and killed between 10am and 1 pm by injection of sodium pentobarbital. Liver samples were harvested and snap-frozen in liquid nitrogen and stored at −80 °C until mRNA preparation and metabolite and lipid extraction.

### RNA preparation and RNA sequencing pipeline

Total RNA was extracted using the RNeasy RNA Mini Kit (Qiagen, Courtaboeuf, France). mRNA was fragmented and converted to cDNA, which was end-repaired, A-tailed and adapter-ligated before amplification and size selection. The prepared libraries were multiplexed and quality controlled before 51-nt paired end sequencing on an Illumina HiSeq2000 sequencer. RNA-Sequencing raw reads were processed using GenPipe^77^. Briefly, reads were trimmed from the 3’ end to have a Phred score of at least 30 and filtered for a minimum length of 32 bp. Illumina sequencing adapters were clipped off from the reads using Trimmomatic v. 0.36^78^. Filtered reads were aligned to the rat genome reference (downloaded from UCSC) using STAR v. 2.5.3 with 2-passes mode^79^. Read counts of Ensembl genes (version 84) were obtained using htseq-count v. 0.6.1^80^.

Differential expression analyses at gene level were performed using the DESeq2^81^ R Bioconductor package. Briefly, raw count data were imported into DESeq2 and normalized using the median-of-ratios approach to account for differences in library sizes and sequencing depth. Gene-specific dispersion estimates were computed under the assumption of a negative binomial distribution, and a generalized linear model (GLM) was then fitted for each gene to estimate log₂ fold changes between conditions. The Wald test was adopted to assess the significance of the model coefficients, and the returned p-values were further corrected for multiple testing using the Benjamini and Hochberg method^82^. Finally, genes with adjusted p-values below 0.05 were deemed differentially expressed.

Raw RNA sequencing data are available through the GenBank Sequence Read Archive (SRA) under the project reference ERP172758: https://trace.ncbi.nlm.nih.gov/Traces/?view=study&acc = ERP172758.

### Biological pathway analysis

Gene Set Enrichment Analysis (GSEA) was performed for functional analysis of the liver transcriptomes^83^. T-statistics of differential expression with default parameters and 1000 permutations were used. P-values were corrected using a significant level of 25% using the Benjamini-Hochberg (BH) method^82^. The Kyoto Encyclopedia of Genes and Genomes (KEGG) (www.genome.jp/kegg), the Panther (www.pantherdb.org) and the Wikipathways (www.wikipathways.org) databases were used to detect enriched pathways in gastrectomized GK rats^21,22,84^. Over-representation analysis (ORA) was also performed. A significant level of 5% after BH correction for multiple testing of p-values was applied. Both GSEA and ORA analyses were performed using the R package WebGestaltR (version 3).

### Quantitative PCR

Quantitative RT-PCR analyses of candidate genes were performed using SYBR green assays (Life technologies, Saint Aubin, France). We used the housekeeping gene *Hprt1* to normalize relative quantification of mRNA levels. Reactions were run on a qTower^3^ Real-Time PCR Thermal Cyclers (Analytik Jena). Oligonucleotide sequences are given in Supplementary Table S9.

### Metabolome and lipidome profiling

Extraction of metabolites and lipids was carried out with frozen liver samples as previously described^85^. Briefly, liver samples were homogenized in a solvent mixture (methanol:water = 4:1) containing 2-isopropylmalic acid (Sigma-Aldrich, Tokyo, Japan) as an internal standard for GC–MS analysis. After centrifugation, supernatants were collected, mixed with water and chloroform and centrifuged. The upper aqueous phase containing hydrophilic metabolites was collected and evaporated with a vacuum concentrator for gas chromatography-mass spectrometry (GC–MS) analysis and the lower phase containing lipid molecules was applied for lipidome analysis using liquid chromatography-mass spectrometry (LC–MS).

For GC–MS analysis, the dried samples were resuspended with a solution containing methoxyamine in pyridine (Sigma-Aldrich, Tokyo, Japan) and N-methyl-N-trimethylsilyl trifluoroacetamide (GL science, Tokyo, Japan). After centrifugation, supernatants were transferred to a glass vial and subjected to GC–MS measurement with a GCMS-TQ8050 (Shimadzu, Kyoto, Japan) as previously described^85^. Multiple reaction monitoring (MRM) modes were based on the Smart Metabolites Database (Shimadzu, Kyoto, Japan). For semi-quantitative analysis, the area of each metabolite peak was calculated and divided by the area of the internal standard peak.

LC–MS analysis was performed with a Nexera UHPLC system coupled with a triple-quadrupole mass spectrometer LCMS-8060 (Shimadzu, Kyoto, Japan). The aliquot of the lower layer was diluted with acetonitrile and injected into the system. Chromatographic separation was conducted on a TSKgel Amide-80 column (150 × 2.0 mm, 3 mm, Tosoh, Tokyo, Japan) in hydrophilic interaction liquid chromatography mode as described^86^.

For both metabolome and lipidome analyses, peak intensities acquired with GC/LC–MS were auto-scaled (Normalized with mean: 0, and standard deviation: 1). Compounds with at least 2 missing values were excluded from subsequent analyses.

### Statistical analyses

Statistical analyses of physiology and metabolome data were performed using GraphPad Prism version 8.4.0 for Windows (GraphPad Software, USA). Shapiro–Wilk tests were performed to assess the normality of data. Statistics were calculated using two-way ANOVA, two-tailed Student t test or Mann Whitney test. Differences were considered statistically significant with a P < 0.05.

## Supplementary Information

Supplementary Information 1.

Supplementary Information 2.

Supplementary Information 3.

Supplementary Information 4.

Supplementary Information 5.

Supplementary Information 6.

Supplementary Information 7.

Supplementary Information 8.

Supplementary Information 9.

Supplementary Information 10.

Supplementary Information 11.

Supplementary Information 12.

Supplementary Information 13.

Supplementary Information 14.

Supplementary Information 15.

## Data Availability

Data is provided within the manuscript or supplementary information files and is available from the corresponding author upon request. Raw RNA sequencing data are available through the GenBank Sequence Read Archive (SRA) under the project reference ERP172758: https://trace.ncbi.nlm.nih.gov/Traces/?view=study&acc=ERP172758.
