# Supplementary material for: Gastrectomy promoted diabetes remission involves the molecular clock and epigenetic mechanisms in a rat model of lean type 2 diabetes
Source: Sci Rep. 2025 Dec 8;16:96. doi: 10.1038/s41598-025-29273-y (PMC12764555; doi:10.1038/s41598-025-29273-y)
Supplement: Supplementary file 1 — Supplementary Information 1. [file 41598_2025_29273_MOESM1_ESM.docx]

**Legends to Supplementary figures**

**Supplementary Figure S1. Wikipathways differentially enriched in diabetic Goto-Kakizaki (GK) rats following vertical sleeve gastrectomy or sham operation.** Gene set enrichment analysis (GSEA) of the liver transcriptomes of gastrectomized (n=4) and sham operated (n=4) GK rats 90 days post-surgery was used to identify pathways up-or down-regulated using the Wikipathway database. Normalised enrichment scores (NES) indicate positive enrichment (blue bars) or negative enrichment (orange bars). Pathways details can be found at [www.wikipathways.org](http://www.wikipathways.org).

**Supplementary Figure S2. Pathway enrichment in liver transcriptomes in diabetic Goto-Kakizaki (GK) rats following vertical sleeve gastrectomy or sham operation using the Panther database.** Differentially enriched Panther pathways were identified using gene set enrichment analysis (GSEA) of the liver transcriptomes of gastrectomized (n=4) and sham operated (n=4) GK rats 90 days post-surgery. Normalised enrichment scores (NES) indicate positive enrichment (blue bars) or negative enrichment (orange bars). Pathways details can be found at www.pantherdb.org.

**Supplementary Figure S3. Overrepresentation analysis (ORA) of biological pathways in diabetic Goto-Kakizaki rats following vertical sleeve gastrectomy or sham operation.** Significant enrichment ratios of KEGG biological pathways were calculated using liver transcriptome data in VSG (n=4) and sham operated (n=4) GK rats 90 days post-surgery. Details of statistics supporting pathway enrichment are in **Supplementary Table 5**. Details of rno pathways can be found at [www.genome.jp/kegg](http://www.genome.jp/kegg).

**Supplementary Figure S4. Expression of genes involved in inflammation (A) or circadian clock (B) in GK rats following vertical sleeve gastrectomy (VSG) or sham operation.** Gene expression was analysed by quantitative PCR in liver of VSG (n=4) and sham operated (n=4) rats 90 days post-surgery. Data are means ± SEM; n = 4 for each rat group; *p<0.05, **p<0.01, ***p<0.001, ****p<0.0001 significantly different between VSG and sham operated GK rats.

**Supplementary Figure S5. Illustration of the polar expression of genes involved in the molecular clock in comparisons between gastrectomized and sham operated Goto-Kakizaki (GK) rats (A) and between GK and normoglycemic Brown Norway rats (B).** Upregulated genes are shown in red and downregulated genes are shown in blue. Log2 Fold changes and P-values are shown for each gene.
