# Supplementary figures and images for: Gastrectomy promoted diabetes remission involves the molecular clock and epigenetic mechanisms in a rat model of lean type 2 diabetes
Source: Sci Rep. 2025 Dec 8;16:96. doi: 10.1038/s41598-025-29273-y (PMC12764555; doi:10.1038/s41598-025-29273-y)

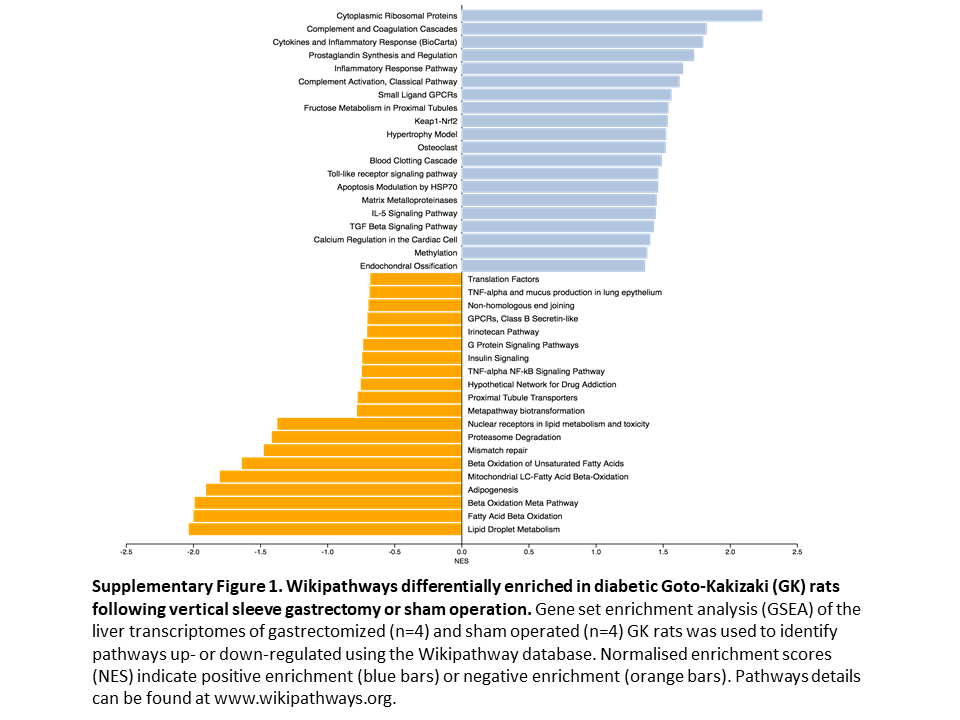

Supplement: Supplementary file 2 — Supplementary Information 2. [file 41598_2025_29273_MOESM2_ESM.tif]

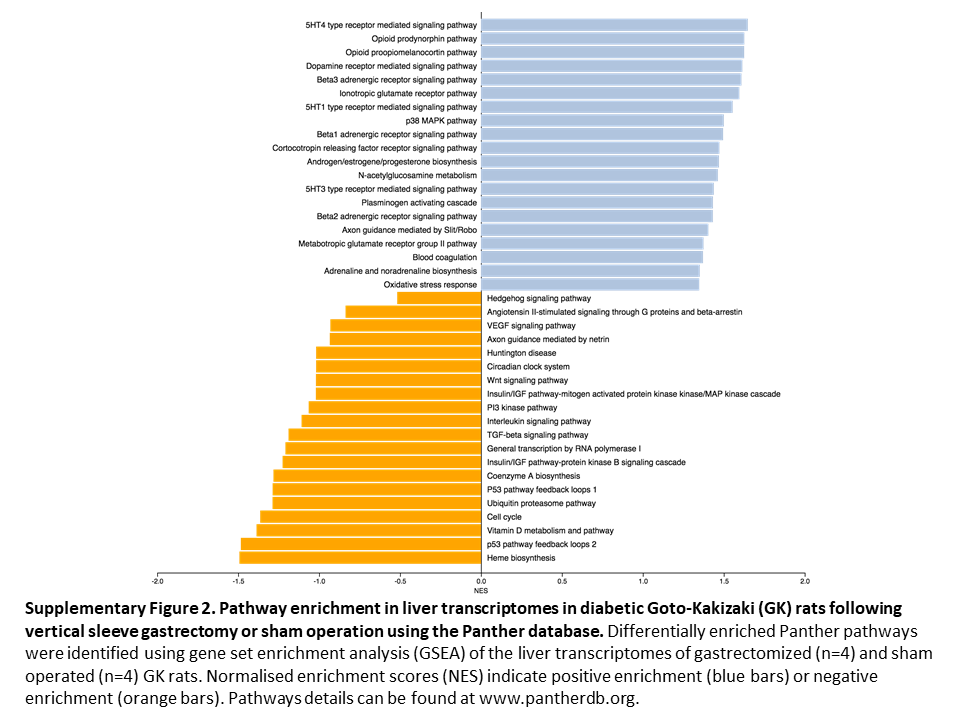

Supplement: Supplementary file 3 — Supplementary Information 3. [file 41598_2025_29273_MOESM3_ESM.tif]

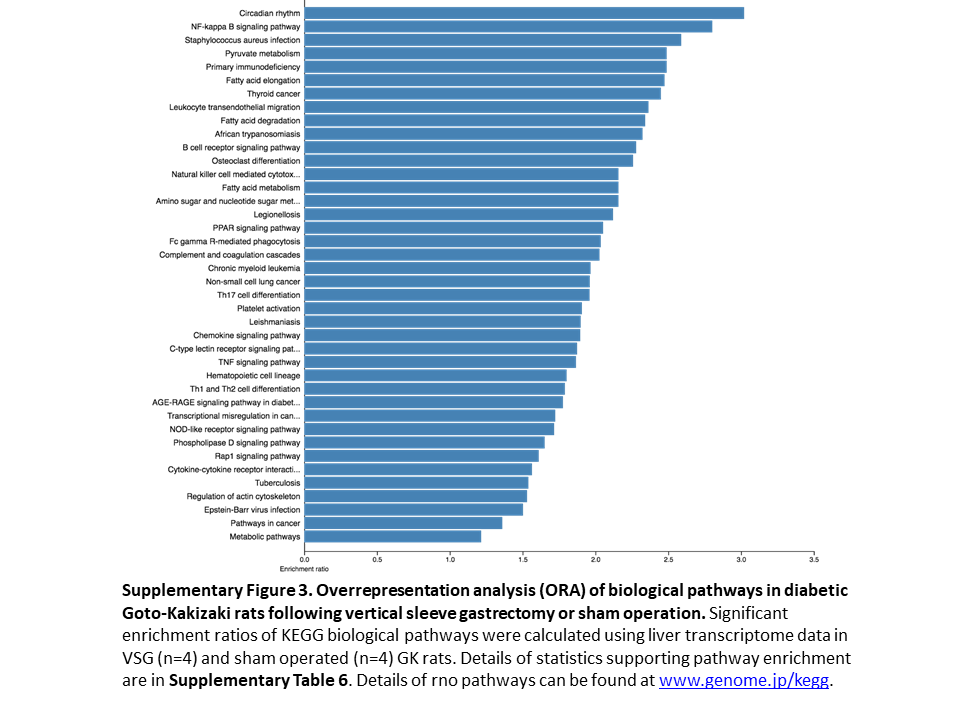

Supplement: Supplementary file 4 — Supplementary Information 4. [file 41598_2025_29273_MOESM4_ESM.tif]

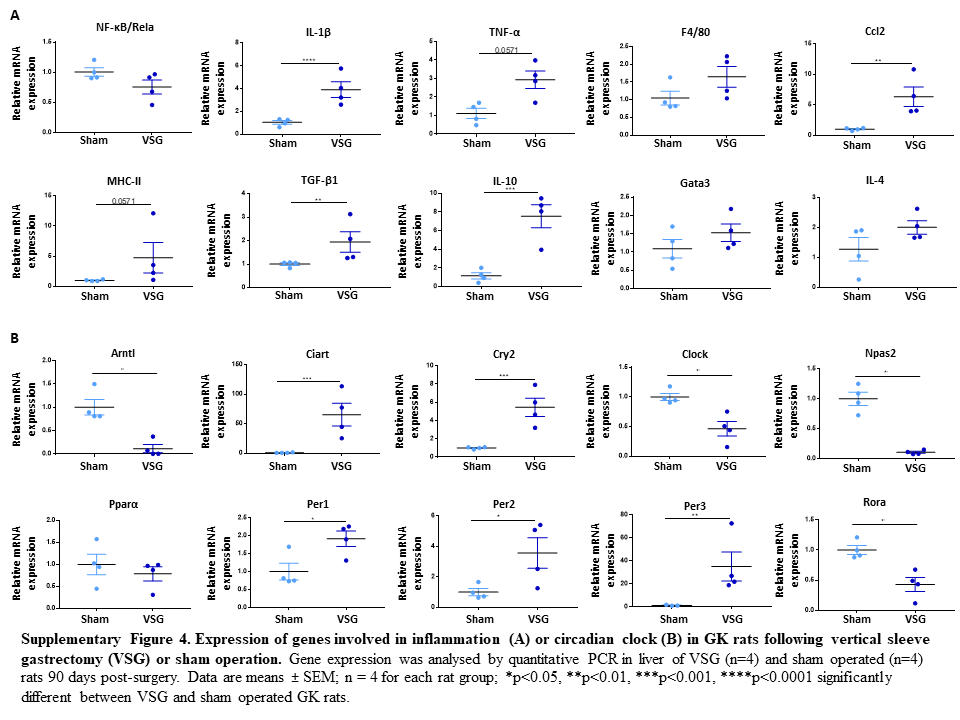

Supplement: Supplementary file 5 — Supplementary Information 5. [file 41598_2025_29273_MOESM5_ESM.tif]

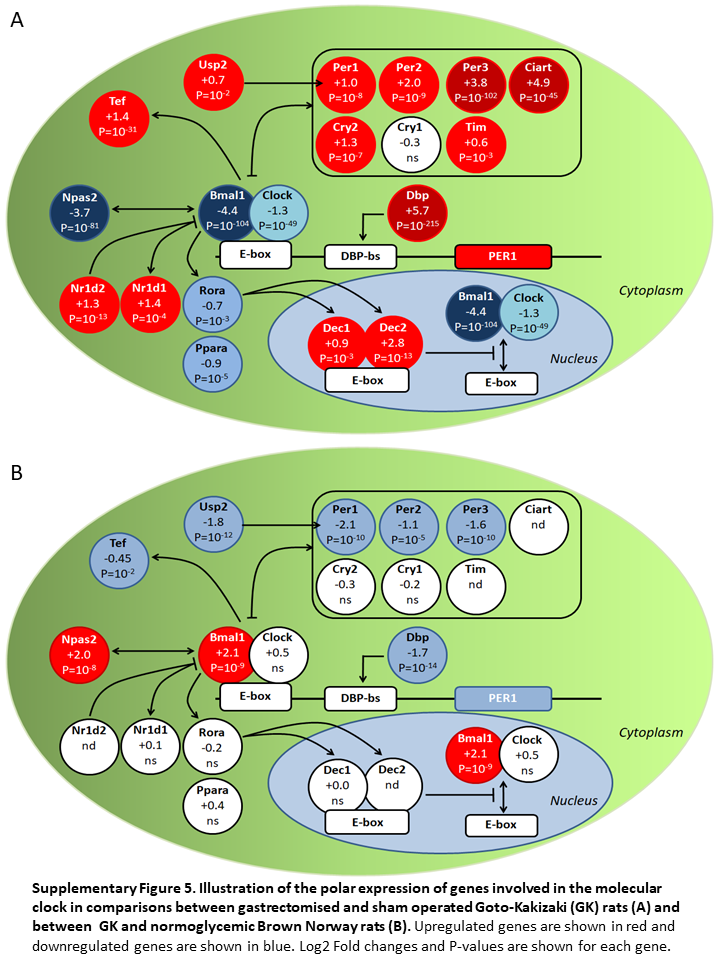

Supplement: Supplementary file 6 — Supplementary Information 6. [file 41598_2025_29273_MOESM6_ESM.tif]
